# Ethanol Affects Network Activity in Cultured Rat Hippocampus: Mediation by Potassium Channels

**DOI:** 10.1371/journal.pone.0075988

**Published:** 2013-11-15

**Authors:** Eduard Korkotian, Tatyana Bombela, Tatiana Odegova, Petr Zubov, Menahem Segal

**Affiliations:** 1 Department of Neurobiology, The Weizmann Institute, Rehovot, Israel; 2 Department of Pharmacognosy, Perm State Pharmaceutical Academy, Perm, Russia; 3 Department of Microbiology, Perm State Pharmaceutical Academy, Perm, Russia; Dalhousie University, Canada

## Abstract

The effects of ethanol on neuronal network activity were studied in dissociated cultures of rat hippocampus. Exposure to low (0.25–0.5%) ethanol concentrations caused an increase in synchronized network spikes, and a decrease in the duration of individual spikes. Ethanol also caused an increase in rate of miniature spontaneous excitatory postsynaptic currents. Higher concentrations of ethanol eliminated network spikes. These effects were reversible upon wash. The effects of the high, but not the low ethanol were blocked by the GABA antagonist bicuculline. The enhancing action of low ethanol was blocked by apamin, an SK potassium channel antagonist, and mimicked by 1-EBIO, an SK channel opener. It is proposed that in cultured hippocampal networks low concentration of ethanol is associated with SK channel activity, rather than the GABAergic receptor.

## Introduction

Alcohol abuse is a major social and medical burden in western society. Extensive neurobiological research has yielded a variety of synaptic and non-synaptic mechanisms affected by exposure to ethanol. This is not surprising, in view of the fact that ethanol can interact with lipids to affect membrane viscosity [1]. There is a growing consensus that a major target for the action of ethanol is the inhibitory GABAergic synapse; as low concentrations of ethanol cause an increase in GABAergic transmission, either directly on synaptic and non-synaptic GABA receptors or via an effect on neurosteroids [2]–[6]. On the other hand, an effect of ethanol on glutamate neurotransmission [7]–[10] as well as cholinergic transmission [11] has also been proposed. While there is an extensive research on possible molecular mechanisms on one end, and behavioral effects on the other, there is a paucity of information on the effects of ethanol on network activity [12] especially in small networks, isolated from the brain. Such a testing system can provide important information on the subtotal action of a drug on local networks, without the confounding effects of extrinsic afferents or extra neuronal loci of action. In the present study we examined the dose-response effects of ethanol on spontaneous activity in networks of cultured hippocampal neurons, and wish to propose that small potassium (SK) channels are a major target of low concentrations of ethanol to affect activity of the network.

## Materials and Methods

### Ethics statement

Animal handling was done in accordance with the guidelines of the Institutional Animal Care and Use Committee (IACUC) of the Weizmann Institute of Science, and the appropriate Israeli law. The Weizmann Institute is accredited by AAALAC. The Weizmann Institutional Animal Care and Use Committee approved this study, conducted with cultured hippocampal neurons. Cultures were prepared as detailed elsewhere [13], [14]. Briefly, rat pups were decapitated on day of birth (P0), their brains removed and placed in a chilled (4°C), oxygenated Leibovitz L15 medium (Gibco) enriched with 0.6% glucose and gentamicin (Sigma, 20 µg/ml). Hippocampal tissue was mechanically dissociated after incubation with trypsin (0.25%) and DNAase (50 µg/ml), and passed to the plating medium consisting of 5% heat-inactivated horse serum (HS), 5% fetal calf serum, and B-27 (1 µl/1 ml) prepared in minimum essential medium (MEM) Earl salts (Gibco), enriched with 0.6% glucose, gentamicin (20 µg/ml), and 2 mM glutamax (enriched MEM). Cells were suspended in 1 ml medium and plated on 12 mm round cover glasses in 24-well plates. Cells were left to grow in the incubator at 37°C, 5% CO_2_ for 4 days, at which time the medium was changed to 10% HS in enriched MEM, plus a mixture of 5′-fluoro-2-deoxyuridine/uridine (FUDR) (Sigma, 20 µg and 50 µg/ml, respectively), to block glial proliferation. Four days later the medium was replaced by 10% HS in MEM.

### Calcium imaging and analysis

Glass coverslips containing the cultures were incubated for 1 hr at room temperature with the standard recording medium (in mM, 129 NaCl,; 4 KCl; 2 CaCl_2_,; 1 MgCl_2_,; 10 Glucose,; 10 HEPES,; pH 7.4, 320 mOsm), containing 2 µM Fluo-4AM (Molecular Probes, USA). Coverslips were placed thereafter on the stage of an inverted Zeiss 510 confocal microscope. Cells were illuminated with a low intensity (0.1–0.2% of nominal) 488-nm light and imaged at a rate of 5–10 frames per second using a 40× oil objective (NA = 1.3, Zoom = 1). Pinhole was adjusted to obtain 3 µm-thick optical slices. Fluorescence variations at 520–550 nm were recorded at room temperature. Transients of [Ca^2+^]i resulting from action potential discharges could be clearly recorded at the imaging rate employed in these experiments. Regions of interest including neuronal somata were measured. Drugs were prepared from frozen stock solutions prior to use, and perfused into the imaging chamber. Only one “naive” field from each culture was used for imaging, so that the total duration of the experiment after the incubation did not exceed 1 hour. The number of fields analyzed matches therefore the number of cultures used. Data were analyzed using Zeiss software for initial image acquisition and measurements of optical density in regions of interest. The homemade program written in MatLab was specified to automatically discriminate events, calculating the time points of spike initiation, peak and decay as well as measuring the rates, durations and synchronization of firing among adjacent neurons. Coefficients of synchronization were calculated by dividing the number of synchronized spikes by the total number of spikes in adjacent neurons. Typically 5–7 neurons were analyzed in a single field of view. ANOVA were used for comparisons among groups. Routinely, experiments were conducted with 4–6 culture glass coverslips, in at least 2–4 litters, unless otherwise stated.

### Electrophysiology

Hippocampal cultures at 13–14 days in-vitro (DIV) were transferred to a recording chamber placed on the stage of an inverted Olympus IX70 microscope and washed with a standard recording medium. Neurons were recorded with patch pipettes containing (in mM): K-gluconate, 136; KCl, 10; NaCl, 5; HEPES, 10; ethylene glycol-bis (beta-aminoethyl ether) N,N,N′,N′-tetra-acetic acid (EGTA), 0.1; Na-GTP, 0.3; Mg-ATP, 1; phosphocreatine, 5; pH 7.2 with a resistance in the range of 5–8 MΩ. When recording spontaneous miniature excitatory postsynaptic currents (mEPSCs), 0.5 µM TTX and 10 µM bicuculline were added to the medium. Neurons were clamped at −60 mV. Signals were amplified with MultiClamp 700B, recorded with pClamp9 (Axon Instruments, Foster City, CA) and analyzed with Minianalysis software (Synaptosoft).

## Results

**Figure 1:**
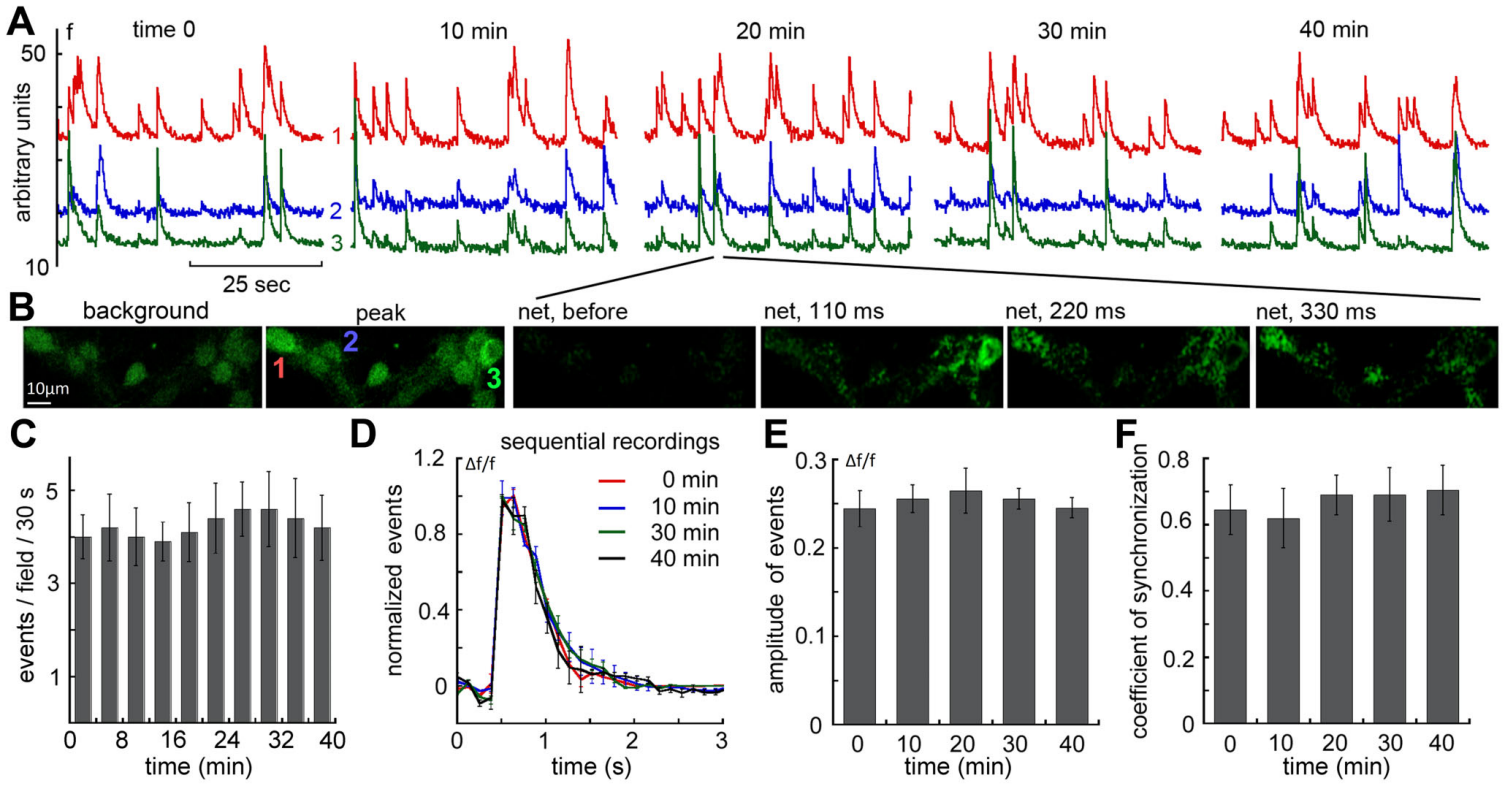
Persistent spontaneous activity in mature cultured hippocampal neurons imaged continuously for over 40 minutes (A). Regions of interest containing three neuronal somata were imaged (snapshots in B, showing background fluorescence, left, peak activity of the three cells marked in color, and net fluorescence at three time points), without a change in rate (C), fluorescence intensity rise time and decay of averaged individual spikes (D), averaged peak amplitudes (E), and coefficient of synchronization among the three imaged neurons (F). These parameters were analyzed automatically using MatLab routines, as indicated in the Methods section.

The first series of experiments was aimed at verifying that the network of neurons in the dish can generate stable activity for up to one hour of recording (Figure 1). Indeed, on the background of low fluorescence intensity, fast ‘spikes’ of [Ca^2+^]i could be recorded simultaneously from several neurons in the field of view. Some, but not all of these events were synchronized. While there were small variations in the inter-spike intervals, overall, the rate of spike discharges was stable across the time of imaging employed in the current study (Fig. 1 C). The events underlying the observed changes in [Ca^2+^]i are likely to represent action potentials evoking calcium influx via voltage gated calcium channels [13]. The current temporal resolution (100–200 msec per frame) does not allow a distinction between single and bursts of action potentials, although the decay of the [Ca^2+^]i surge is fairly standard (Fig. 1 D), and most of the transients emitted in a given cell are of about the same magnitude/decay time, indicating their being triggered by single spikes. This is an important parameter, since it could change following drug exposure (see below). In addition to the rate of spike discharges, we also calculated the spike amplitudes (Fig. 1E), decay kinetics (Fig. 1D) and coefficient of synchronization, which indicates the magnitude of synchronized bursts among all the recorded neurons in the field of view (Fig. 1F). These two parameters were also rather stable across an hour of a recording session.

### Ethanol effects

**Figure 2:**
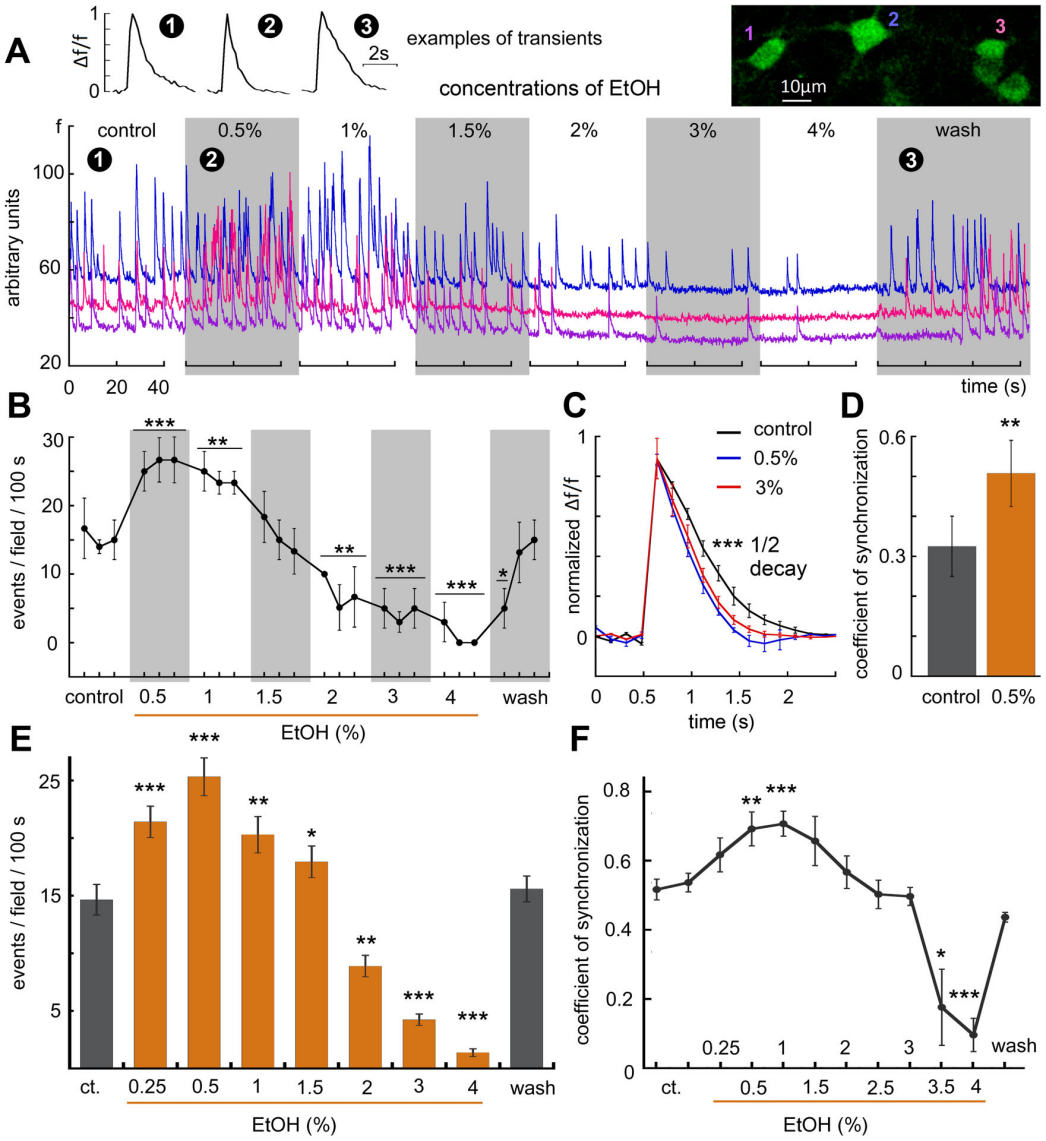
Effects of increasing concentrations of ethanol (EtOH) on spontaneous network activity. A, sample illustration of an individual experiment, comprising ROI's of three cells (different colors) in the field of view (Illustrated in color on top right), demonstrating that exposure to 0.5% EtOH caused an increase in the frequency of the network bursts, whereas higher concentrations (2–4%) caused a gradual suppression of activity, along with a reduction in baseline fluorescence. Notably, washout of the EtOH caused a return to baseline activity without apparent long lasting effects of the drug. Expanded scales of some individual spikes are shown on top left: numbers denote the time in the experiment where the samples were taken. B–D, a summary of the rates of activity analyzed from the experiment shown in A; low EtOH caused a rise in frequency of events, whereas the high concentration caused suppression of activity. C, the rise time of individual spikes was the same for the different concentrations, whereas the decay was significantly faster in the presence of EtOH. D, the coefficient of synchronization, showing the relative proportion of synchronized events, was significantly larger during exposure to low concentrations of ethanol. E, averaged results of 27 experiments comprising 162 neurons, where low concentrations (0.25%) as well as all higher concentrations of EtOH were used, to show significant dose-dependent changes in spontaneous network bursts. F, same, with the coefficient of synchronization shown for the different concentrations of EtOH.

Increasing concentrations of ethanol were applied to cultures while these were imaged continuously (Figure 2A). Low concentrations (0.5%, equivalent to 80 mM) caused an increase in rate of spike discharges in the cultured neurons (Figure 2 B, 5 cover glasses imaged, total of 34 cells, a 95% increase in firing rate, from 13.5±1.45 to 26.4±1.84 spikes per 100 seconds, p<0.0004, by paired t-test). This change was associated with the speeding up of the decay of the calcium transients (1/2 decay = 0.442±0.065 seconds in control, down to 0.319±0.041 in presence of 0.5% ethanol, p<0.0001). Interestingly, the low ethanol also caused a significant increase in the coefficient of synchronization (Figure 2D). Higher concentrations of ethanol (2–4%) caused a marked decrease in firing rates, down to complete cessation of discharges. Upon washout of ethanol, there was a gradual return to baseline firing rates, indicating that the short (1–2 minute) exposure to even the high ethanol concentration (4%, near 0.65 M) did not produce a long lasting detrimental effect on the activity of the recorded neurons.

In another series of experiments even a lower concentration of ethanol (0.25%, ∼40 mM) caused a significant, albeit smaller rise in rate of spike discharges, as well as a significant increase in the coefficient of synchronization (Figure 2F). The minimum drug dosage still able to produce a statistically significant increase in the firing rate was about 0.09% (∼15 mM, data not shown). Interestingly, the higher concentration of ethanol caused a decrease in synchronization among the recorded neurons. Once again, washout of ethanol restored both the firing rate and the synchronization among the recorded neurons (Figure 2 E, F).

### Ethanol and synaptic currents

**Figure 3:**
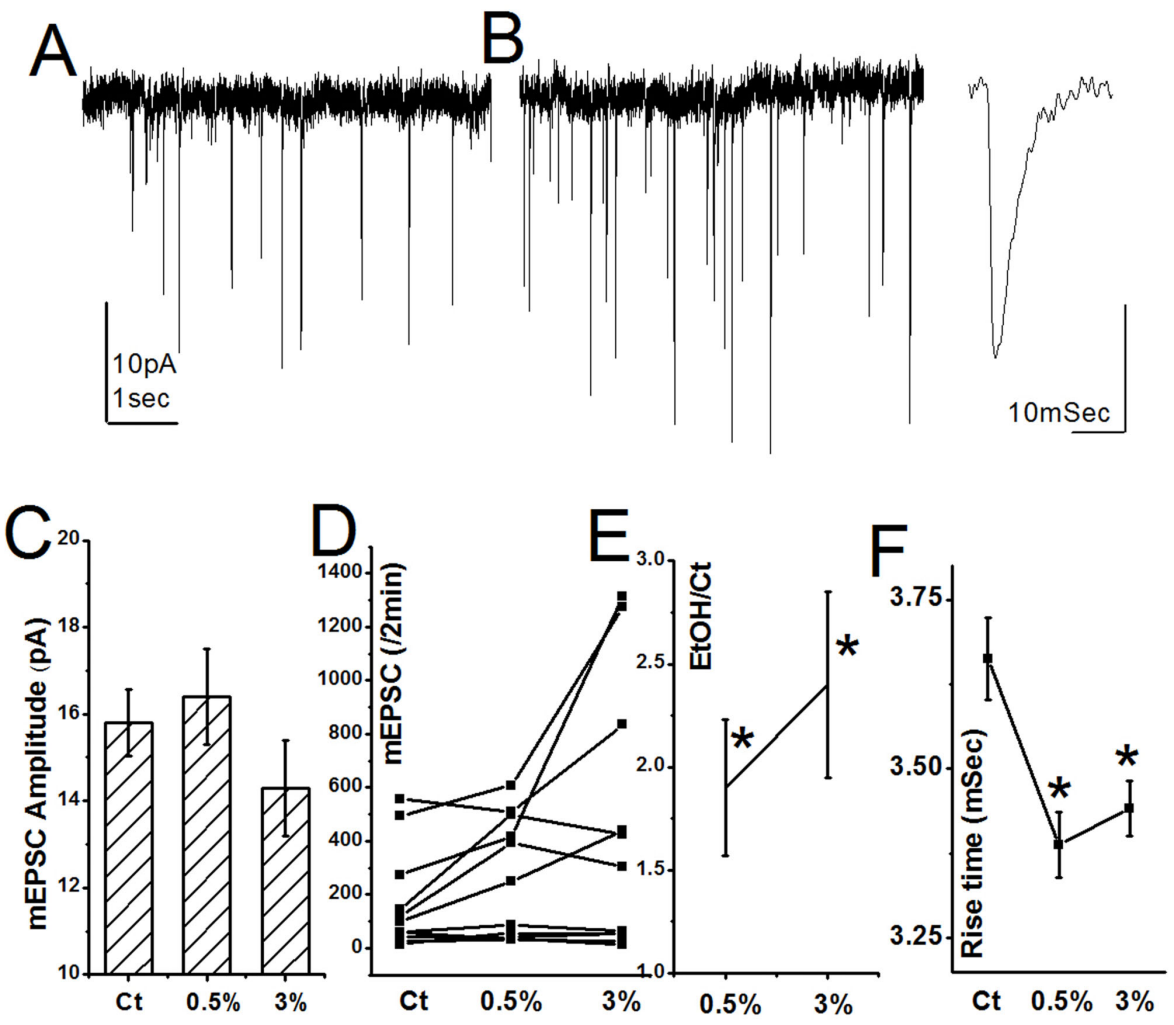
Electrophysiological effects of EtOH in patch-clamped cultured hippocampal neurons. A&B, sample illustrations of synaptic current recordings in a voltage clamped neuron recorded near its resting membrane potential of −60 mV. Spontaneous activity was recorded before (A) and after exposure to 0.5% EtOH (B). Insert on the right illustrates the time course of an individual mEPSC. C. Averaged mEPSC amplitudes (in pA) of the same group of 11 neurons, before and during exposure to 0.5% and 3% EtOH. Significant differences were found between the three means. D, rates of mEPSCs plotted in individual neurons before, during 0.5 and 3% EtOH, respectively. E, the same as in D, with means of changes from control condition plotted as a function of the different EtOH concentration. A significant increase in rates was found (p<0.05). F, mean rise time of the individual mEPSCs was recorded under the different conditions. While the change in rise time was small, it was highly significant (p<0.01).

The possible synaptic locus of effect of ethanol was studied using patch clamp recording from 11 neurons, one cell in each culture glass in three different dissections. Recording was made in the presence of TTX, to block action potential discharges, and bicuculline, to suppress possible effects of ethanol on inhibitory synapses. Under standard conditions, a baseline activity was recorded for two minutes, followed by application of 0.5% ethanol, recording for 2 minutes, application of 3% ethanol, and recording again for two additional minutes. The pipette resistance was stable across the recording period. The mean mEPSC size was not different significantly in the three recording conditions (15.8±0.75, 16.3±1.14 and 14.2±1.08 pA in control, 0.5% and 3% Ethanol, respectively (Figure 3A–C). The frequencies of mEPSC were variable among the three conditions, in most of the neurons mEPSC frequencies went up after exposure to ethanol (Figure 3E) and if the individual changes between the recording conditions were analyzed, a significant rise was found (1.9±0.33 and 2.4±0.45 times baseline rates (Figure 3E). Interestingly, the rise time of the mEPSCs was significantly faster during exposure to both concentrations of ethanol (Figure 3 F). These experiments indicate that ethanol may have both a presynaptic locus of action (expressed as a rise in frequency of mEPSCs) as well as a postsynaptic locus, expressed as a change in kinetics of mEPSCs.

### Ethanol and GABA

**Figure 4:**
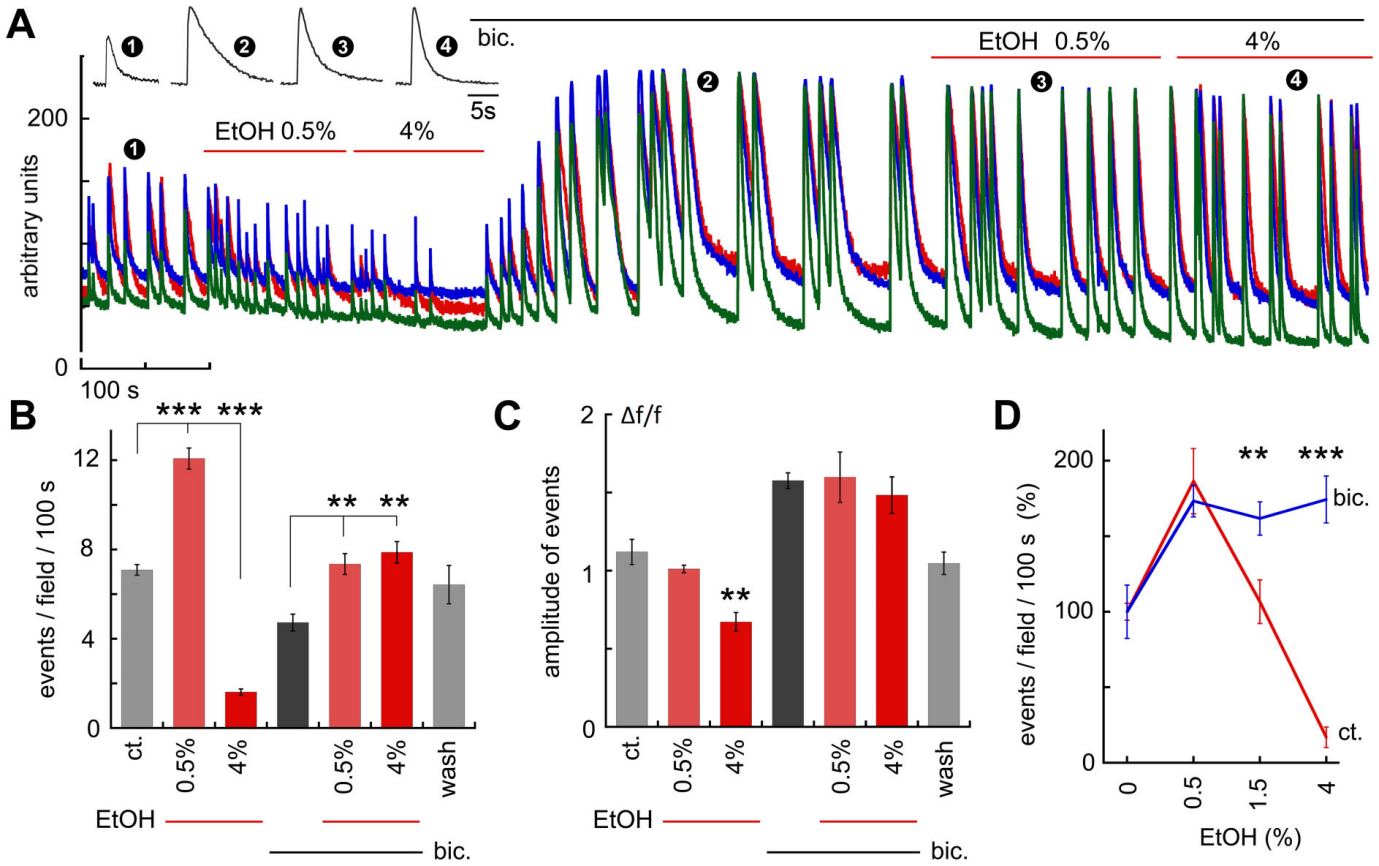
The facilitating action of low concentration of EtOH on network activity was not mediated by an action at a GABAergic receptor. A, sample illustration of the effects of low and high EtOH in control and following blockade of GABA receptors with bicuculline (bic, upper line). While the facilitating action of low EtOH was sustained in presence of bic, the suppressing action of high EtOH was blocked by bicuculline. Sample traces are shown on upper left, numbers denote the time where these traces were sampled. B&C, bar graphs, summarizing 5 experiments to show that while bicuculline reduced the frequency of network bursts (B), it increased the magnitude of individual bursts (C). In presence of bicuculline, the number of events was significantly larger during low EtOH. D, a summary diagram of the effects of different concentrations of EtOH in absence and presence of bicuculline.

The possible involvement of GABAergic neurotransmission in either the low concentration increase in spike rates, or the high concentration blockade of activity was tested in 5 experiments (32 cells in total). Initially, the cultures were exposed to low ethanol, which produced the typical increase in firing rate, and then to 4% ethanol, which blocked activity. The culture was then perfused with 10 µM bicuculline, a GABA-A receptor antagonist. Bicuculline produced a 2–3 fold increase in size of the spikes (Figure 4A,C), which was associated with a decrease in the rate of spike discharges (Figure 3B) and a marked increase in spike duration (Figure 4A insert).In presence of bicuculline, 0.5% ethanol was still able to increase spike frequency significantly (n = 5 fields, 32 cells, frequency went up from 4.8±0.34 to 7.4±0.27, p<0.0001). Interestingly, high ethanol (1.5 and 4%) was no longer able to suppress spontaneous network burst (Figure 4B, D), indicating that the high concentration of ethanol indeed acts by enhancing GABA receptor activity, but the increase in activity following low concentration of ethanol does not seem to involve GABAergic neurotransmission.

### Ethanol and SK channels

**Figure 5:**
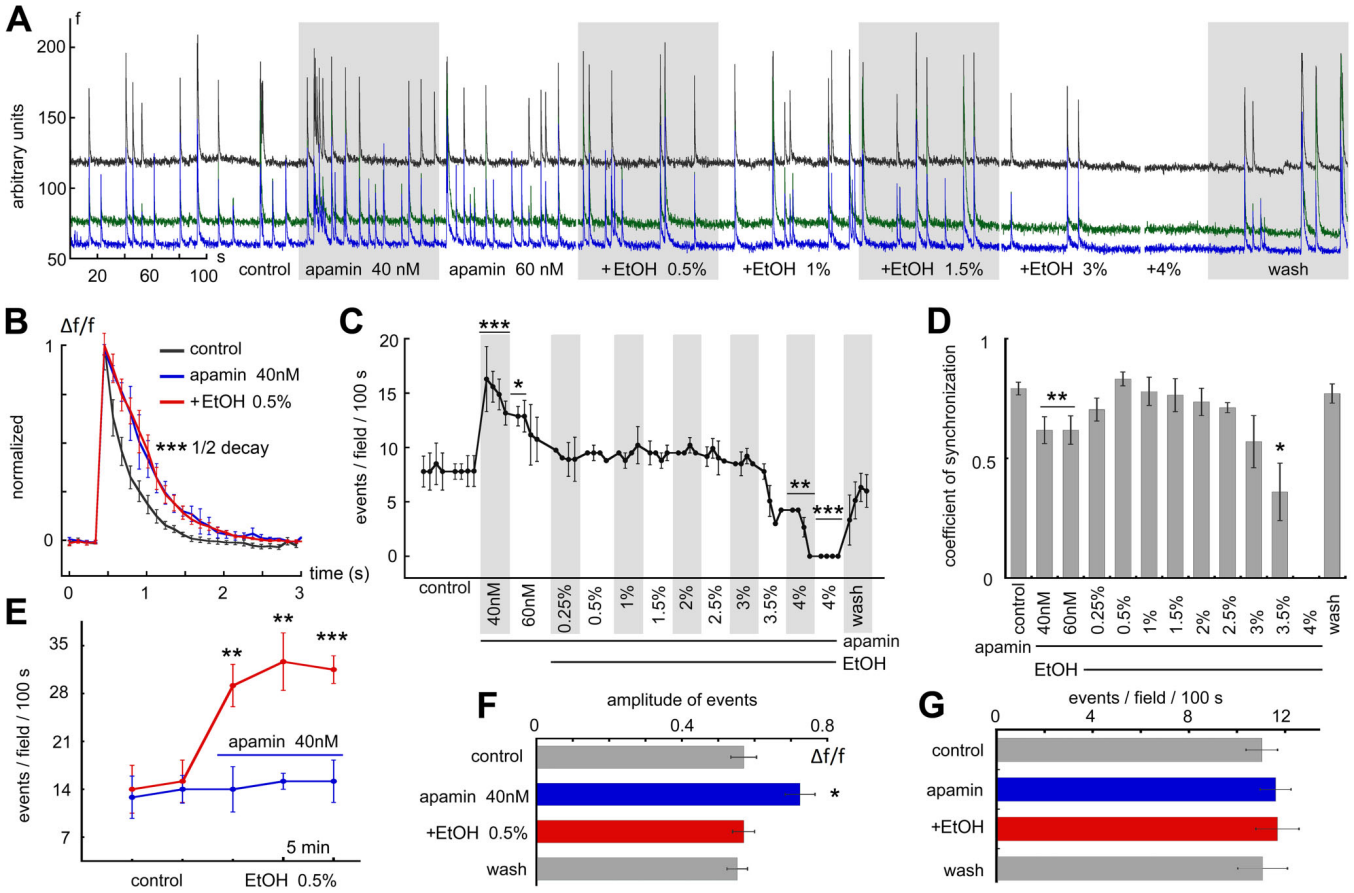
The BK channel antagonist, apamin, blocked the facilitating action of EtOH on network bursts. A, sample recording of 3 neurons in the field of view, demonstrating that apamin at low concentration (40 nM) produces a transient facilitatory action on network activity. In the presence of apamin, when activity returned to baseline, EtOH was no longer able to increase the rate of network bursts. Nonetheless, the higher concentration of EtOH was still able to suppress network activity. Interestingly, washout of both apamin and EtOH caused a return of baseline activity. B, the duration of individual bursts was significantly prolonged by apamin, as expected from its blocking action of SK channels, and this effect was not reversed by EtOH. C, summary of 9 experiments (44 cells), where exposure to apamin generated a transient increase in frequency of network bursts, followed by a return to baseline activity, during which no further increase was produced by low concentrations of EtOH. Higher concentrations of EtOH caused a suppression of activity, regardless of the presence of apamin. D, bar graph presentation of the drug effects on the coefficient of synchronization. While apamin reduced synchronization, EtOH did not change this parameter compared to control values. E, changes in rate of network bursts by EtOH, in absence and presence of apamin. As indicated in the illustrations above, apamin suppressed the facilitation of activity produced by EtOH. F, summary of the drug effects on amplitudes of the spontaneous events. Apamin caused a rise in amplitude of the spikes (in addition to the prolongation of the duration of the spikes, seen in B), but this action was antagonized by low EtOH. G, summary of the sustained effects of apamin and EtOH on frequency of network bursts. As seen above, presence of apamin prevented the rise in frequency of events caused by EtOH.

**Figure 6:**
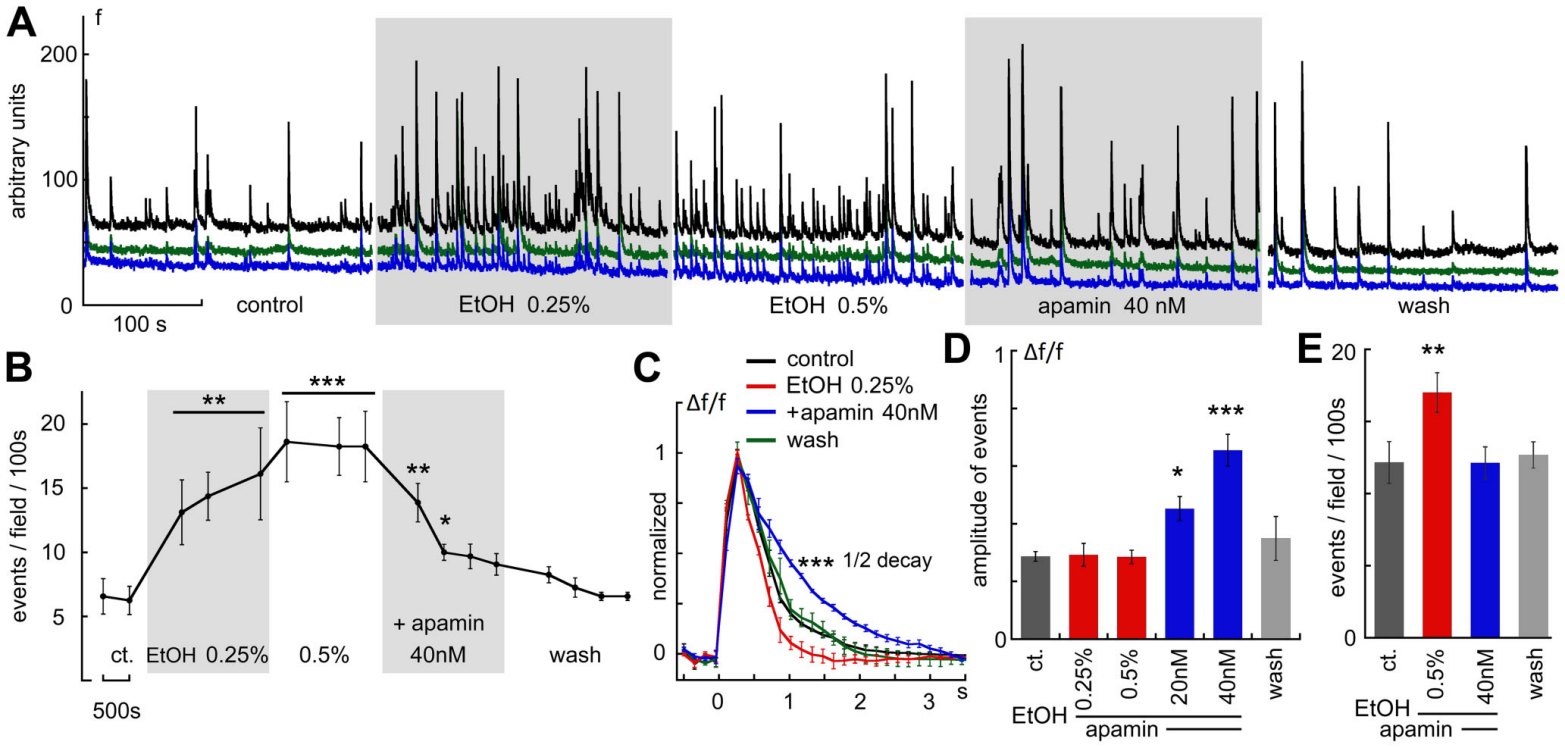
Apamin suppresses the elevated network activity produced by exposure to low EtOH. A, the progress of the experiment; initially the culture was exposed to 0.25% and then to 0.5% EtOH, which produced a sustained rise in network activity. Further, the addition of 40 nM apamin caused a return to baseline network activity. B, summary of the experiment shown in A, the rate of activity was plotted as a function of time/drug exposure. C, time course of averaged spikes, before, during and after exposure to the two drugs. While EtOH caused shrinkage of the spike duration, apamin reversed and prolonged spike duration. D, amplitudes of the averaged individual spikes were not affected by low concentrations of EtOH, but were significantly increased by apamin, in correlation with the prolongation of the duration of the spikes (C ). Finally, a bar graph summary of the effects of EtOH on frequency of events, as seen in B, before and during exposure to apamin (E). A significant increase in rate of firing was abolished by apamin, as seen before.

earlier studies proposed that ethanol may affect potassium channels of primarily the small conductance (SK) species [15]–[17]. Using a selective antagonist for the SK channels, apamin, we explored the role of these channels in the effects of ethanol on cultured network activity (Figure 5). Application of low concentration (40 nM) apamin to the culture caused a significant but transient two fold rise of in the spike discharge rate, as well as an increase in the averaged spike duration (Fig. 5B) and size (Figure 5F, 8 experiments, 51 neurons). However, continuous exposure to apamin (Figure 6E, one experiment, 7 neurons), and even an increase in concentration of the peptide to 60 nM (Figure 5C), did not sustain the increase in the rate of spike discharges, which eventually returned to baseline rate (5 experiments, 33 neurons). On the other hand, the decay time of the spikes was significantly slowed down, as expected from a blocker of potassium channels (Figure 5B). However, in the presence of apamin, low concentration of ethanol (0.25–0.5%) was no longer able to cause an increase in network activity (Figure 5C, G, 8 experiments, 51 neurons), and was also unable to reduce the delayed recovery of the averaged spike back to baseline. In contrast, high concentrations of ethanol could still suppress baseline activity down to zero (Figure 5A, C, D), indicating that these effects are not mediated by an action at the SK channel. Once again, washout of the drugs caused a complete recovery of spike discharge rate (Figure 5C, D).

To verify that the interaction between apamin and ethanol is not due to the sequence of drug application, ethanol was applied first in another series of experiments (Figure 6, 5 experiments, 38 cells), to produce the typical sustained rise in rate of spike discharges. Following was the application of apamin, which blocked the effect of ethanol on spike rates, while it caused an increase in spike sizes as well as their duration (Figure 6 B, C). The summary of 9 experiments (44 neurons, Figure 6D, E) indicates that caused a significant increase in amplitudes of the spikes, while it suppressed the effects of low EtOH on rate of spike discharges. These experiments indicate that regardless of the order of application, apamin blocked the effects of ethanol on spike rates, while having a sustained effect of its own on spike size and decay.

**Figure 7:**
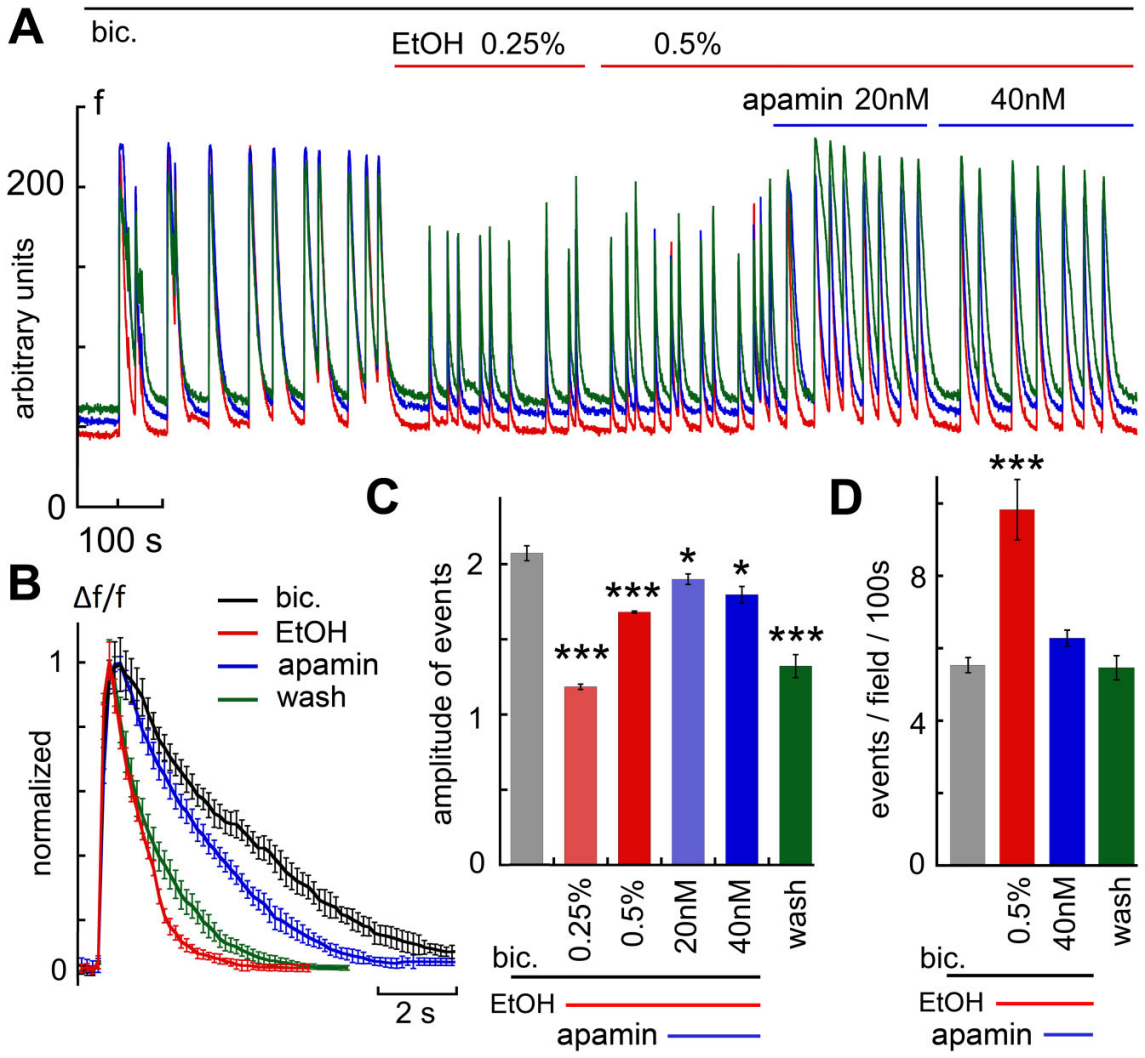
The effects of apamin on EtOH were not mediated by an interaction with the GABAergic system. A, experiments were conducted in presence of bicuculline, which caused the formation of larger and sparse network bursts. The magnitudes of the bursts were reduced by EtOH, and their frequency was increased (summarized in C&D, 6 experiments, 49 cells). Apamin then caused an increase in the size of the events but reduced their frequency back to control level. The presence of EtOH caused a significant reduction in the duration of the bursts (B), but apamin prolonged them back to the level seen in presence of bicuculline.

To examine possible interactions between apamin and the GABAergic system, the effects of apamin on ethanol action was studied in presence of the GABA-A receptor antagonist bicuculline. As seen above (Figure 4), bicuculline caused a large increase in spike size, which was associated with a marked prolongation of spike duration. Also, presence of bicuculline did not deter the facilitating action of ethanol on spike rate (Figure 7A, single experiment, D, summary of 6 experiments, 49 neurons), while it significantly reduced spike height (Figure 7C). In presence of apamin, spike frequency was significantly reduced (Figure 7D), while spike height climbed gradually back to its original size (Figure 7C). These results indicate that while bicuculline and apamin may share an effect on spike height and duration, most likely due to activation of different synaptic and channel properties, they differ in their ability to block the effects of ethanol.

**Figure 8:**
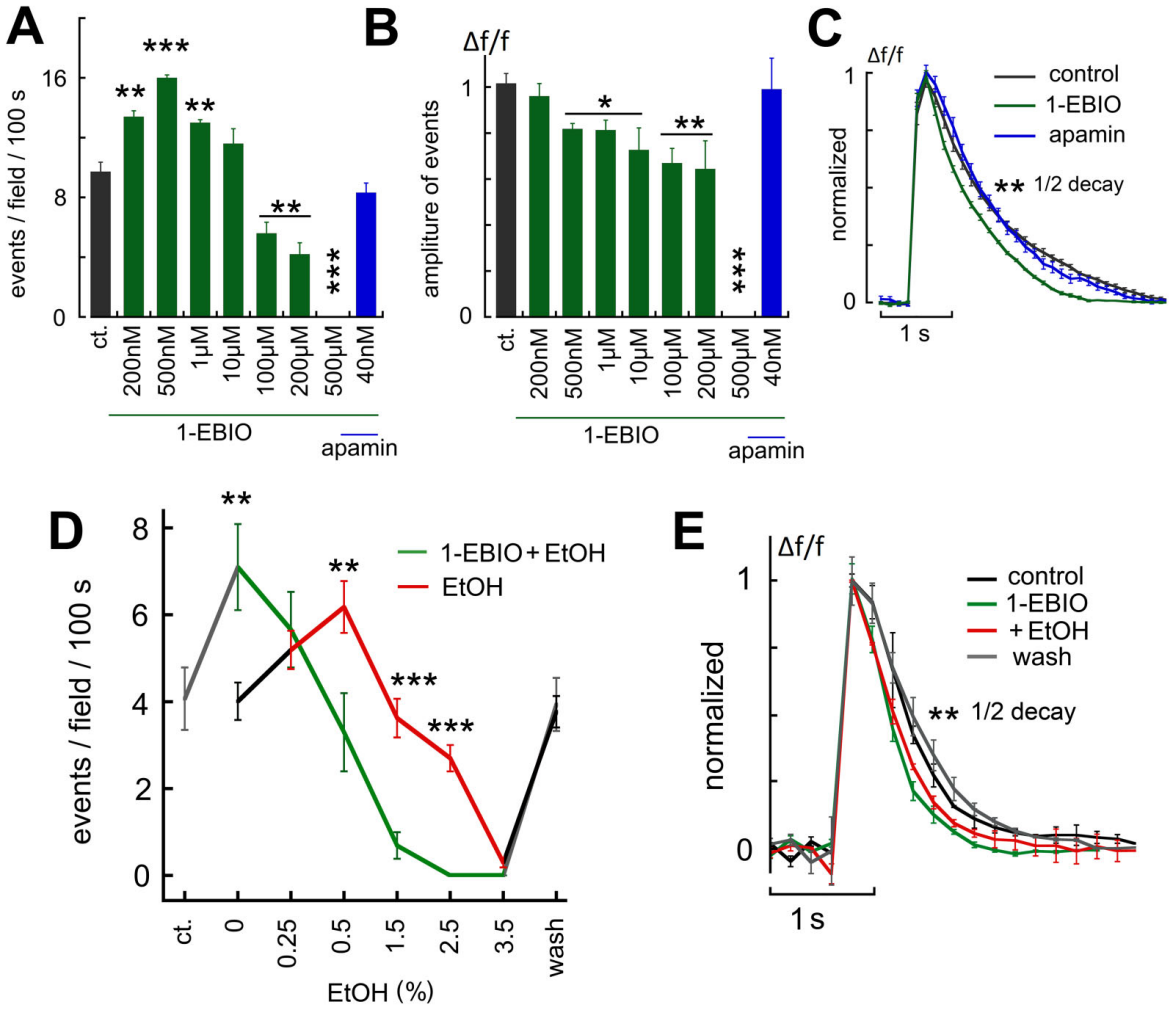
The SK channel opener 1-EBIO mimicked the effects of EtOH on spontaneous network activity. A, at low concentrations (200 nM to 500 nM) 1–EBIO caused an increase in frequency of network spikes, while at higher concentrations(200–500 µM) it suppressed network activity, in line with its role as a K channel opener. Apamin blocked the effects of 1-EBIO (right column). B, the averaged amplitudes of the network spikes was monotonically reduced by increasing concentrations of 1-EBIO, with no apparent biphasic response, as was the case in A. C, averaged rise time of the individual bursts was the same in control and in presence of 1-EBIO, but the decay was faster in presence of the drug, and apamin blocked this effect. D&E, the combined effects of 1-EBIO and EtOH on frequency of network spikes (D) and spike decay time (E) in the cultured neurons (4 experiments). Low (500 nM) 1-EBIO caused a significant increase in rate of spike discharges, an effect that was mimicked by low EtOH. However the combined exposure to low EtOH and 1-EBIO enhanced significantly the suppressing action of EtOH, seen with higher concentration of the drug. In addition, 1-EBIO caused shrinkage of the spike duration, and the effect was not further amplified by EtOH (E).

Finally, if indeed the effects of ethanol are mediated by activation of SK channels, it was important to compare the effects of ethanol to those of the SK channel opener 1-EBIO (1-Ethyl-1,3-dihydro-2H-benzimidazol-2-one). At the concentrations used by others (200 µM [17], 1-EBIO produced a marked suppression of spontaneous activity, likely to be caused by activation of potassium channels (Figure 8A, 15 experiments, 81 neurons). Surprisingly, low concentrations of 1-EBIO (200–500 nM) produced a 51.2% rise in rate of spike discharge (Figure 8A). This was associated with the speeding up of the decay of the averaged spike, an opposite effect than that produced by apamin, and similar to that produced by low concentration of ethanol (Figure 8C). These effects of 1-EBIO were completely reversed by 40 nM apamin, as expected (Figure 8A, B).

If indeed low concentration of ethanol acts like 1-EBIO, the synergistic action of both agents may result in enhancement or reversal of the action of either of them, since both agents at high concentration suppress spontaneous network activity. To examine these possibilities, the cultures were exposed to different concentrations of EtOH (Figure 8D, E). There was no apparent facilitation of the enhanced spontaneous spike discharge by 1-EBIO. In fact, at a concentration that did not produce any effect on its own (1.5%) there was a significant suppression of activity, by the addition of 1-EBIO (Figure 8D).

**Figure 9:**
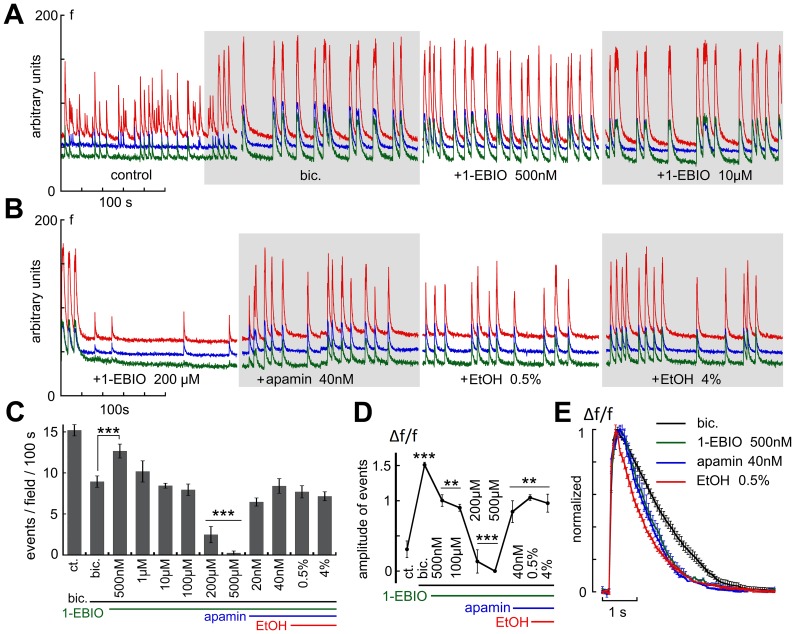
The effects of 1-EBIO were not mediated by an action at the GABA receptors. A&B, sample experiment where bicuculline was applied first, to cause generation of larger and fewer network spikes. In the presence of bicuculline, low concentration of 1-EBIO (500 nM) was still able to increase the rate of network spikes, whereas a higher concentration of the drug reduced their frequency, down to a complete silencing of the network. Apamin blocked the effects of 1-EBIO in the presence of bicuculline. Under these conditions, EtOH was no longer able to modify network activity (C). D, changes in amplitudes of spikes as a function of drug exposure. Drugs are denoted by bars below the average amplitudes of the spikes. E, changes in duration of the spikes as a function of bicuculline (causing a prolongation of the spikes). Addition of 1-EBIO caused shrinkage of the spike duration, whereas apamin prolonged spike duration, and EtOH caused further shrinkage of the spikes.

As the silencing action of 1-EBIO on network activity is similar to that produced by high EtOH which enhances GABAergic neurotransmission, we explored this possibility by applying the drug in presence of bicuculline. Under these conditions, bicuculline caused an enlargement of the size of network spikes as well as a reduction in their frequency (as in Figure 5). In presence of bicuculline, 1-EBIO at low concentration was still able to speed up network spike frequency (Figure 9A, C), as well as shorten the duration of the individual spikes (Figure 9E). Unlike EtOH, higher concentration of 1-EBIO was still able to suppress network activity (Figure 9C), an effect that was blocked by apamin, which also blocked the facilitatory effects of low concentration of EtOH.

## Discussion

The present studies examined the effects of different concentrations of ethanol on spontaneous network activity of dissociated cultures of rat hippocampus. Activity was assessed through the imaging of transient rises of [Ca^2+^]i accompanying action potential discharges in the network. Under standard conditions, persistent activity can be recorded for hours. The rate of spike discharges is a sensitive measure of the subtotal of effects on either synaptic or soma/dendritic loci of action of the tested drug. Naturally, drugs of multiple loci of action can produce complex effects on network activity. Ethanol at low concentrations produced a persistent rise in network activity, which was strikingly different from that produced by high ethanol concentration, which blocked activity altogether. The latter effect was mediated by an enhancing action of GABAergic neurotransmission, as it was blocked by bicuculline, a GABA-A antagonist, and involved an increase in the rate of spontaneous inhibitory synaptic currents (data not shown). The enhancing effect of low ethanol was not affected by bicuculline, but was sensitive to apamin, an SK channel antagonist. It was accompanied by a rise in the rate of spontaneous mEPSCs in the recorded neurons. Finally, ethanol effect was quite similar to that produced by low concentration of 1-EBIO, an SK channel opener, further supporting the indication that it may facilitate the activation of SK channel.

The present results confirm and extend previous results on the involvement of SK channels in the action of ethanol [15], [16], [17], [18], [19], but they differ in several respects from previous studies. It must be said that other studies used different test systems, which perhaps are not as sensitive as the current one to low concentrations of drugs. On the other hand, the current results do not provide direct test for the involvement of specific ion channels or receptors, and further studies are needed to explore at the more biophysical level the action of the different drugs.

Nonetheless, we demonstrate an effect of a very low concentration of 1-EBIO, below those used in other studies: 300 µM [19] or 400 µM [17], to demonstrate facilitation rather depression of spontaneous network activity. As our current studies are designed to examine the effects of a drug, ethanol in this case, on the behavior of an entire network, there are several mechanisms that can be affected by the drug subtly, and the outcome of these additive effects will result on a way that is not predicted by a single site of action. One such possibility is that 1-EBIO opens potassium channels more in interneurons than in pyramidal neurons, resulting in a reduction in inhibitory tone, and a subsequent increase in network activity. This is less likely, in view of the enhancing action of 1-EBIO on spontaneous activity even in the presence of bicuculline, which blocks the inhibitory synapses. At any rate, experiments with low concentration of 1-EBIO are required to be conducted at the single cell level in order to examine possible mechanisms underlying this effect of 1-EBIO. One possible supporting evidence for the involvement of SK channels in the facilitating action of ethanol on network bursts is in the change in kinetics of mEPSCs observed in our study. This has to be further explored and substantiated.

Another issue of incongruence between the current experiments and earlier ones is the assumed involvement of GABAergic neurotransmission in the effect of ethanol. In earlier studies, even low concentration (40–80 mM) of ethanol is proposed to act by facilitating the action of GABA at either synaptic or non-synaptic receptors [5]. Such facilitation should result in suppression of spontaneous activity, opposite to what we find using similarly low ethanol concentrations. Furthermore, this facilitative action is resistant to bicuculline, indicating that it is independent of GABA receptors. Interestingly, another recent study [3] was unable to demonstrate a significant effect of ethanol on spontaneous GABAergic synaptic activity in cultured hippocampal neurons, and so the verdict concerning GABA role in ethanol effect is not entirely clear.

One of the confounding factors in comparing results from different studies is the tissue where the cells were recorded from. Wakita et al. [5], [20] reported that ethanol enhances GABAergic action in isolated hippocampal neurons form young animals. Carta et al [21] found that very low concentration of ethanol (10 mM) blocks the facilitating action of kainic acid on spontaneous IPSCs in hippocampal slices and this is mediated by a presynaptic interaction with GABAergic terminals. Yet another mechanism has been proposed to mediate the effect of ethanol on freshly isolated supraoptic neurons, which involve modulation of voltage gated calcium channels [22]. Thus, different mechanisms may be activated by different concentrations of ethanol, under different testing conditions. Some of these reported mechanisms may be involved in the facilitating action of ethanol seen in the present studies, and further experiments will elucidate these possible routes of action.

One of the possible sites of action is the excitatory synapse. Our results indicate that an acute effect of low ethanol is to enhance the frequency of spontaneous miniature excitatory synaptic currents, as well as speed up their rate of rise. The latter effect may be mediated by a change in SK channels on dendritic spines, where these synapses are located. Interestingly, our results are somewhat different from those of Basavarajappa et al [7] who also found a lack of effect on mEPSC amplitudes, but they report on a suppression of mEPSC frequencies. This effect was recorded at 15–20 minutes after onset of exposure to ethanol, at times needed to activate the endocannabinoid receptors, whereas our results are found at much shorter times (2–4 minutes). We do not exclude a later reduction in mEPSC frequencies, and this will be subject to further experimentations. The heterogeneity of effects of ethanol may indicate that different cell types may respond differently to a low concentration of ethanol, as indicated in another recent study conducted with a similar culture of cortical neurons [23].

At the behavioral level, low concentrations of ethanol, calculated to be similar to the ones used here, can produce a state of enhanced activation and loss of inhibitory control, whereas higher dose produce suppression of behavior and loss of consciousness and eventual death. These are similar to the dose-response relations seen in the current study, except that even the complete suppression of activity with high concentration of ethanol (4%) is washable, and does not lead to persistent damage to the recorded neurons.

Further electrophysiological experiments where properties of individual neurons in a network will be analyzed, may contribute to the understanding of the cellular and molecular mechanisms responsible for the pronounced action of alcohol on human behavior.
