# Correction: Ethanol Affects Network Activity in Cultured Rat Hippocampus: Mediation by Potassium Channels

**DOI:** 10.1371/annotation/49d33906-ea34-403b-b477-84e82cb87c81

**Published:** 2013-12-03

**Authors:** Eduard Korkotian, Tatyana Bombela, Tatiana Odegova, Petr Zubov, Menahem Segal

A funding organization was incorrectly omitted from the Funding Statement. The Funding Statement should read: "1/2012; Wezimann711458, Grant of The Ministry of Education and Science of Perm Krai, Russian Federation entitled “International Research Groups of Scientists (IRG)” URL:http://minobr.permkrai.ru/activity/wu/. This study was also supported by a grant from the Perm Territory Government, Russian Federation (International Research Groups, 2011). The funders had no role in study design, data collection and analysis, decision to publish, or preparation of the manuscript." 

