# Correction: Ethanol Affects Network Activity in Cultured Rat Hippocampus: Mediation by Potassium Channels

**DOI:** 10.1371/annotation/4777fd66-ffe6-4d3d-afbf-be0165764e9c

**Published:** 2014-01-29

**Authors:** Eduard Korkotian, Tatyana Bombela, Tatiana Odegova, Petr Zubov, Menahem Segal

An error occurred in the Figure 5 title. The sentence refers to "The BK channel", but it should refer to "SK."

